# Multiple hepatic sclerosing hemangiomas: a case report and review of the literature

**DOI:** 10.1186/s40792-018-0468-6

**Published:** 2018-06-19

**Authors:** Kyohei Yugawa, Tomoharu Yoshizumi, Noboru Harada, Takashi Motomura, Norifumi Harimoto, Shinji Itoh, Toru Ikegami, Yuji Soejima, Yoshihiko Maehara

**Affiliations:** 1 Department of Surgery and Science, Graduate School of Medical Sciences, Kyushu University, Maidashi 3-1-1, Higashi-ku, Fukuoka, 812-8582 Japan; 2 Department of Hepatobiliary and Pancreatic Surgery, Gunma University, Showa-machi 3-39-22, Maebashi-shi, Gunma 371-85111 Japan

**Keywords:** Hepatic, Multiple, Giant, Sclerosing hemangioma, Pathology

## Abstract

**Background:**

Hepatic sclerosing hemangioma, a very rare benign tumor, is characterized by fibrosis and hyalinization occurring in association with degeneration of a hepatic cavernous hemangioma. Such atypical hemangiomas can be diagnosed incorrectly as primary or metastatic malignancies based on imaging characteristics. We present herein a rare case of giant and multiple hepatic sclerosing hemangiomas that are difficult to differentiate from hepatic malignancies and review the relevant literature.

**Case presentation:**

The patient was a 48-year-old male who was found to have multiple hepatic tumors and a giant tumor (67 × 53 mm) superior to the inferior vena cave by an abdominal ultrasonography during a routine medical examination. The patient was referred to our hospital for further evaluations and diagnosis of the multiple hepatic tumors. Dynamic CT showed low-density tumors in the delayed phase suggestive of membrane-covered lesions. EOB-MRI demonstrated a mass with low-signal intensity mass on T1-weighted images and areas of high-signal intensity on T2-weighted images and a hypointense mass in the hepatobiliary phase, which showed high intensity on DWI-based ADC map. FDG-PET showed no accumulation of [^18^F]-FDG. A provisional diagnosis of multiple scirrhous hepatocellular carcinomas was made on the basis of these imaging studies, and caudate lobectomy of the liver and partial hepatectomy of S2 and S6 were performed. Histopathological examination showed that the tumors were composed of various sized irregularly dilated vessels with some blood thrombi, inflammatory cell infiltration, fibrous and hyalinized sclerotic or myxomatous stroma, resulting in a diagnosis of multiple hepatic sclerosing hemangiomas.

**Conclusions:**

Differentiation of multiple sclerosing hemangiomas from other hepatic malignant tumors, such as intrahepatic cholangiocarcinoma, metastatic liver cancer, and scirrhous hepatocellular carcinoma characterized by abundant fibrous stroma, is difficult because the radiological findings are very similar. Inclusion of hepatic sclerosing hemangioma in the differential diagnosis of multiple liver tumors could enable optimal management; this possibility is important to consider before planning invasive therapies.

## Background

Hepatic sclerosing hemangioma, a very rare benign tumor, is characterized by fibrosis and hyalinization occurring in association with degeneration of a hepatic cavernous hemangioma [1]. Even with recent developments in radiological modalities, it is difficult to diagnose preoperatively because it is benign and extremely rare, and its radiological features resemble those of other liver tumors such as hepatocellular carcinoma, metastatic liver cancer, and intrahepatic cholangiocarcinoma [2, 3]. We here report a case of multiple hepatic sclerosing hemangiomas that was diagnosed preoperatively as multiple scirrhous hepatocellular carcinomas. We also review relevant published reports, especially focusing on radiological findings of hepatic sclerosing hemangioma.

## Case presentation

A previously healthy 48-year-old male was found to have multiple hepatic tumors and a giant tumor (67 × 53 mm) superior to the inferior vena cave by abdominal ultrasonography during a routine medical examination. He consulted a general physician for further evaluations and had been referred to us because plain CT confirmed multiple tumors, including a giant tumor, in the liver. A laboratory workup on admission showed that total bilirubin (0.6 mg/dL) and albumin (4.2 g/dL) concentrations were within their normal ranges, whereas aspartate aminotransferase (37 IU/L), alanine aminotransferase (70 IU/L), alkaline phosphatase (176 U/L), and gamma-glutamyl transpeptidase (170 IU/L) concentrations were mildly increased. Tumor markers, including alpha-fetoprotein (2.9 ng/ml), protein induced by vitamin K absence or antagonist-II (11 mAU/ml), and carcinoembryonic antigens 19-9 (4.0 IU/L), were within normal limits.

**Fig. 1:**
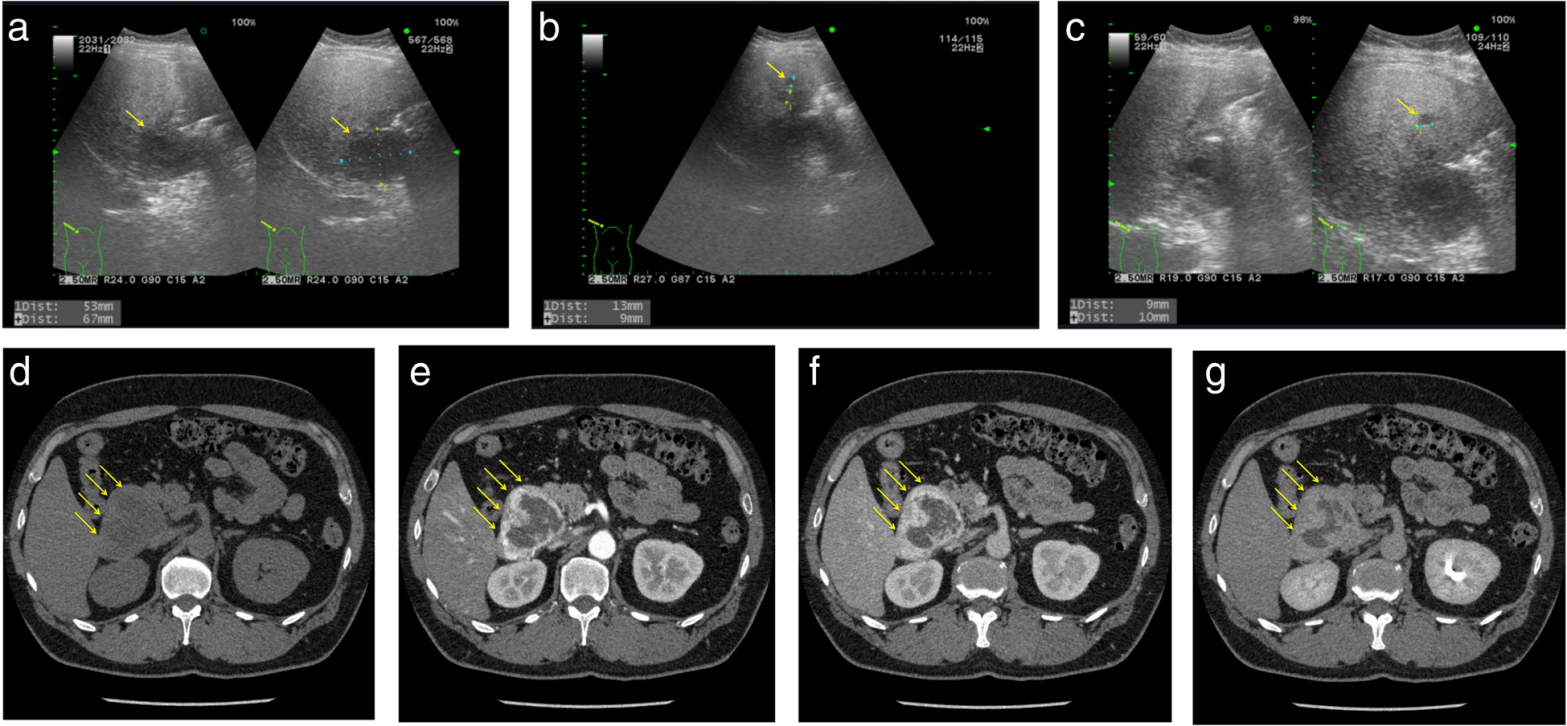
Abdominal ultrasonography (US) and contrast-enhanced abdominal computed tomography (CT). US images showing well-defined, hypoechoic masses in segments 1 (**a**), 2 (**b**), and 6 (**c**). Pre-contrast CT scan image showing a low-density 65-mm mass with an irregular margin in the caudate region (**d**). Contrast-enhanced CT scans images revealing peripheral early ring enhancement in the arterial phase and internal heterogeneous enhancement in the delayed phase (**e**–**g**)

**Fig. 2:**
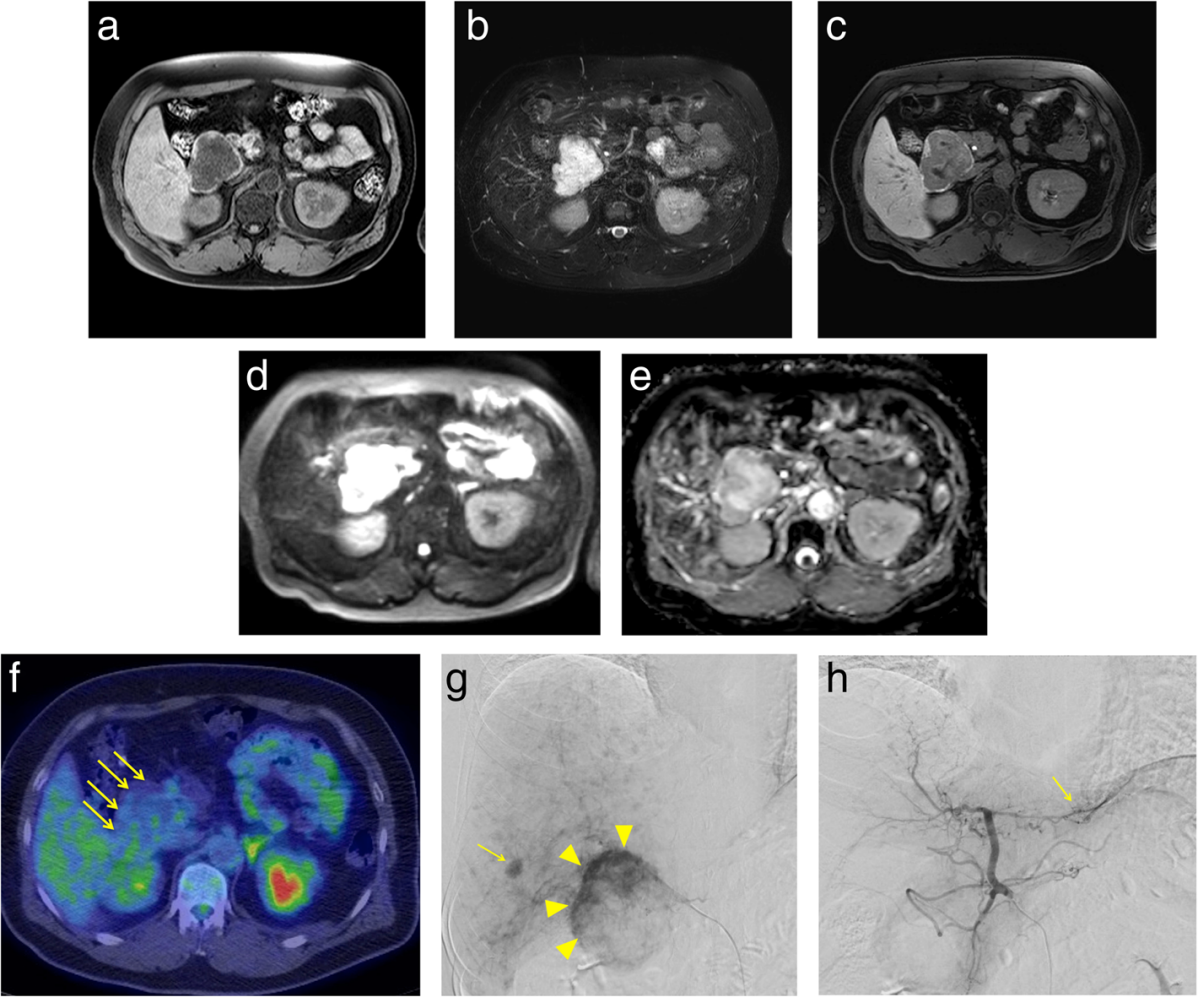
Magnetic response imaging (MRI), [^18^F]-fluorodeoxyglucose positron emission tomography (FDG-PET) and hepatic arteriography. MR images showing low-signal intensity on T1-weighted images (**a**) and high-signal intensity on T2-weighted images (**b**). EOB-MRI revealed a hypointense mass in the hepatobiliary phase (**c**). The ADC mean of DW-MRI in the mass was 2.33 × 10^−3^ mm^2^/s (**d**, **e**). There is no accumulation of [^18^F]-FDG (**f**). Hepatic arteriography showing hypervascular masses in S1, S6 (**g**), and S2 (**h**) of the liver; there is no pooling

Abdominal ultrasonography (US) revealed well-defined, hypo echoic masses in segment 1 (S1) (67 × 53 mm in diameter), S6 (13 mm), and S2 (9 mm) in the liver (Fig. 1a–c). Abdominal dynamic CT revealed a low-density 65-mm diameter mass with an irregular margin in plain, peripheral early ring enhancement in the arterial phase, and internal heterogeneous enhancement in the delayed phase (Fig. 1d–g). Gadolinium-ethoxybenzyl-diethylenetriamine pentaacetic acid-enhanced magnetic response imaging (EOB-MRI) demonstrated a low-signal intensity mass on T1-weighted images. This mass contained several high-signal intensity areas on T2-weighted images. EOB-MRI also revealed a hypointense mass in the hepatobiliary phase (Fig. 2a–c). Furthermore, it showed higher intensity compared with the normal liver parenchyma on DWI with a high *b* value of 1000 (Fig. 2d). Its ADC value was 2.11 × 10^−3^ mm^2^/s (peripheral area) and 2.45 × 10^−3^ mm^2^/s (central area), and the ADC mean was 2.33 × 10^−3^ mm^2^/s. It was described as a high-intensity mass on the ADC map (Fig. 2e). There was no accumulation of [^18^F]-FDG on FDG-PET (Fig. 2f). Hepatic arteriography revealed hypervascular masses in S1 (65 mm in diameter), S6 (16 mm), and S2 (9 mm) of the liver, with no pooling (Fig. 2g, h). In addition, gastroscopy and colonoscopy showed normal findings.

**Fig. 3:**
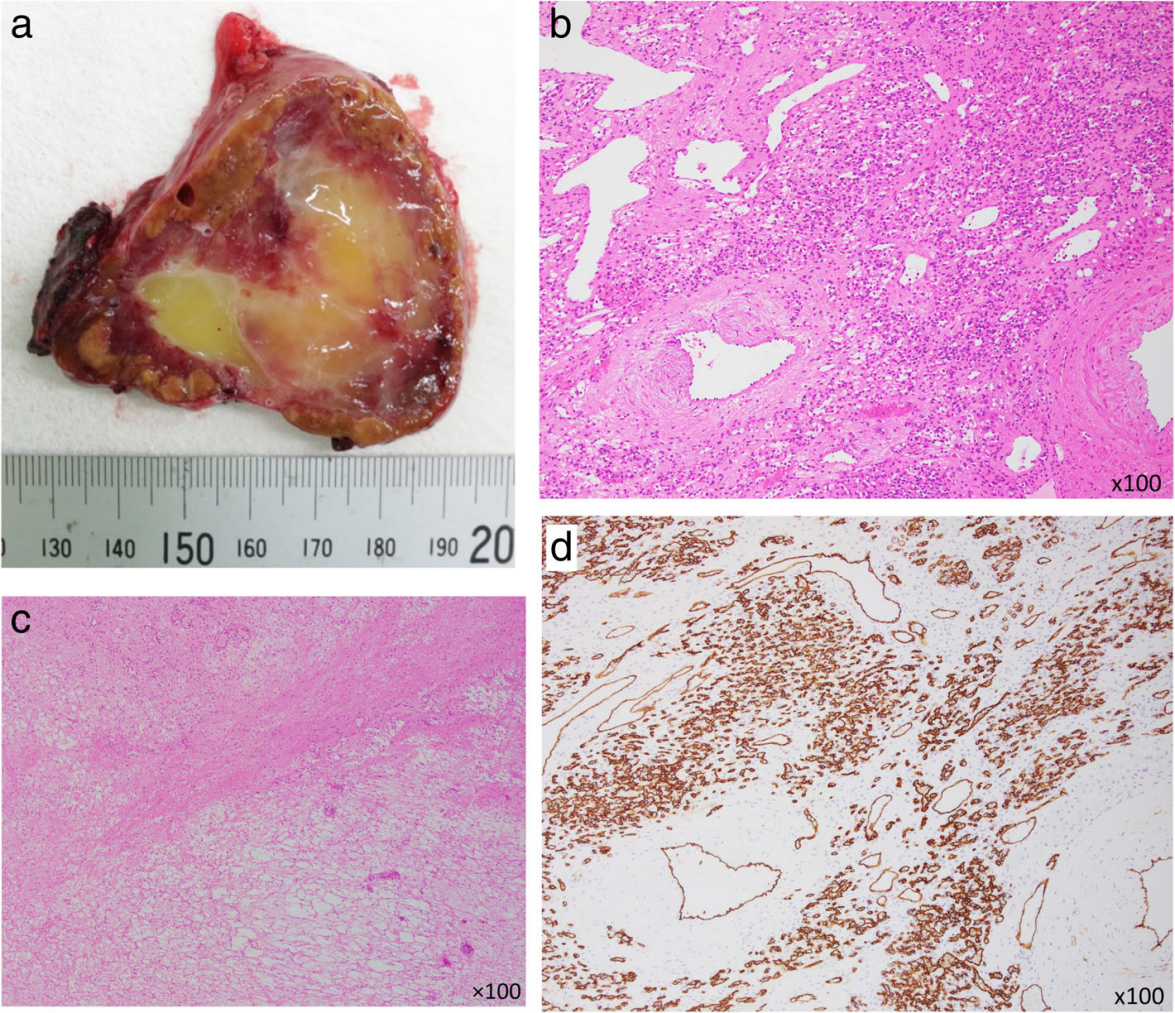
Resected specimen. The cut surface of the tumor in S1 reveals a 55 × 56 mm, well-demarcated, homogeneous, yellowish-white solid mass (**a**). The tumors were composed of various sized irregularly dilated vessels with fibrous and hyalinized sclerotic stroma (hematoxylin and eosin staining, H&E; **b**, **c**), CD34 positive vascular components (**d**)

On the basis of these imaging findings, a preoperative diagnosis of multiple scirrhous hepatocellular carcinomas characterized by abundant fibrous stroma was made, and caudate lobectomy of the liver and partial hepatectomy of S2 and S6 were performed. The resected specimen contained a 55 × 56 mm yellowish white solid mass. The cut surfaces of the tumor were elastic and soft with smooth margins (Fig. 3a).

Histopathological examination showed that the tumors were composed of various sized irregularly dilated vessels with some blood thrombi, inflammatory cell infiltration, fibrous and hyalinized sclerotic or myxomatous stroma (Fig. 3b, c). On immunohistochemistry staining, these vascular endothelial cells were positive for CD34, suggesting sclerosing hemangioma (Fig. 3d). The patient tolerated the procedure well and was discharged on postoperative day14. The patient gave consent for publication of details of his case.

### Discussion

Hemangiomas are the commonest benign hepatic tumors, being found in up to 7% of autopsies in one series [4]. Hemangiomas have a predilection for women in a ratio of 5:1. They are characteristically discovered incidentally during abdominal imaging in individuals aged 40 to 50 years [5]. Hepatic sclerosing hemangioma, first reported by Shepherd et al. in 1983 [6], is a rare type of hepatic hemangioma composed of abundant acellular hyalinized tissue in which small vessels are occasionally seen. Another study reported finding them in only two of 1000 autopsies [7]. Hepatic sclerosing hemangiomas are caused by degenerative changes such as thrombus formation, necrosis, and scar formation in hepatic cavernous hemangiomas; however, the mechanism(s) for these degenerative changes has not yet been determined [1]. Makhlouf and Ishak compared the findings in sclerosed hemangioma and sclerosing hemangioma [8]. They asserted that recent hemorrhages and hemosiderin deposits rich in mast cells are present in sclerosing hemangiomas, whereas fibrosis, increased elastic fibers, and dystrophic or psammomatous calcifications with decreased numbers of mast cells are characteristic of sclerosed hemangiomas [8].

These pathological changes can result in the radiological features on CT and MRI being atypical of hemangioma; thus, these lesions can be diagnosed as malignant tumors such as intrahepatic cholangiocarcinoma, metastatic liver cancer, and hepatocellular carcinoma. We searched for “hepatic sclerosing hemangioma” in PubMed and ICHUSHI, a bibliographic database established in 1903 and being updated by the Japan Medical Abstracts Society, and identified 41 patients, including our patient, the characteristics of which we here summarize.

Concerning imaging studies, Miyamoto et al. [9] summarized the imaging findings in 41 hepatic sclerosing hemangiomas. Their average diameter was 41.8 mm, ranging from 10 to 145 mm. Abdominal US showed hyperechoic masses in 10 patients and hypoechoic masses in 14. Plain CT generally showed a low-density mass, whereas dynamic CT showed ring enhancement, resembling metastatic liver cancer or intrahepatic cholangiocarcinoma, in 31 of 40 (78%) reported cases. MRI showed low-intensity signals in 28 of 30 (93%) reported cases on T1-weighted images and high-intensity signals in 25 of 30 (83%) reported cases on T2-weighted images. FDG-PET showed no accumulation of [^18^F]-FDG in six patients who underwent this procedure [9]. The radiological features revealed by dynamic CT and MRI resembled those of hepatic malignancies. Thus, there were indeterminate imaging features, some imaging findings pointing to intrahepatic cholangiocarcinoma, metastatic liver cancer, or scirrhous hepatocellular carcinoma (ring enhancement, hyperintensity on T2 with hypointensity on T1 imaging). Generally, imaging findings of ring enhancement on dynamic CT is less typical of classical hepatocellular carcinoma than of metastatic liver cancer or intrahepatic cholangiocarcinoma. In recent years, there have been a small number of reports in which DWI and DWI-based ADC were successfully used to differentiate sclerosing hemangioma from liver malignancies. The ADC values for hepatic hemangiomas are reported to be higher than those of malignant liver tumors due to restricted water diffusion from high cellular density [10, 11]. In our case, the ADC mean was 2.33 × 10^−3^ mm^2^/s, and the mass showed higher than the normal liver parenchyma with a high *b* value of 1000 on DWI. It was described as a high intensity mass on an ADC map, indicating that possibility of the sclerosing hemangioma, used as a diagnostic aid to detect. FDG-PET showed no accumulation of [^18^F]-FDG, it suggested the possible benign character of this sclerosing hemangioma. However, it should be noticed that negative predictive value of FDG-PET for primary hepatocellular carcinoma is less than 50% with sensitivity over 80% [12]. Therefore, we selected hepatic resection as a first-line strategy for the management of tumors with unknown potential.

Histopathologically, the cut surfaces of our patient’s resected tumors corresponded with cross-sectional CT images; the dynamic enhancement pattern is related to the vascular spaces component and the central areas of low density on the arterial and delayed phase to the sclerotic component, which on examination were yellowish-white with many hyalinized areas with poor vessels and fibrous changes [13, 14]. Additionally, our patient’s serum tumor markers, including AFP and PIVKA-2, were within normal limits. If these multiple tumors had been hepatocellular carcinomas, concentrations of these markers may have been high. Thus, it also suggests that the multiple tumors were not hepatocellular carcinomas.

Confirmation of our provisional diagnosis would have required obtaining a liver biopsy. Although a fine needle biopsy can differentiate a sclerosing hemangioma from hepatocellular carcinoma, this procedure can potentially lead to rupture or seeding of hepatocellular carcinoma. Fine needle biopsies should only be performed to confirm inoperable hepatocellular carcinoma because seeding of tumor in the needle tract has been reported in 1–3% of cases [15]. We suspected that our patient’s lesions were scirrhous hepatocellular carcinomas because of the imaging features. In addition, it was considered that the lesion protruding into the intraperitoneal cavity rupture was at risk of rupture. The likelihood of malignancy and risk of rupture resulted in our decision to perform hepatic resection on our patient.

## Conclusions

This case report illustrates the atypical imaging appearance of sclerosing hemangioma, and these imaging may cause confusion and a diagnosis of scirrhous hepatocellular carcinoma. Although sclerosing hemangiomas are not frequently encountered in clinical practice, they are included in the differential diagnosis of hepatic malignant tumors, such as intrahepatic cholangiocarcinoma, metastatic liver cancer, and scirrhous hepatocellular carcinoma.

## Abbreviations

CT: Computed tomography
EOB-MRI: Gadolinium-ethoxybenzyl-diethylenetriamine pentaacetic acid-enhanced magnetic response imaging
FDG-PET: Fluorodeoxyglucose positron emission tomography
US: Ultrasonography

## References

[1] Cheng HC, Tsai SH, Chiang JH, Chang CY (1995). Hyalinized liver hemangioma mimicking malignant tumor at MR imaging. AJR Am J Roentgenol.

[2] Takayasu K, Moriyama N, Shima Y, Muramatsu Y, Yamada T, Makuuchi M (1986). Atypical radiographic findings in hepatic cavernous hemangioma: correlation with histologic features. AJR Am J Roentgenol.

[3] Ros PR, Lubber PR, Olmsted WW, Morillo G (1987). Hemangioma of liver: heterogeneous appearance on T2-weighted images. AJR.

[4] Dietrich CF, Mertens JC, Braden B, Schuessler G, Ott M, Ignee A (2007). Contrast-enhanced ultrasound of histologically proven liver hemangiomas. Hepatology.

[5] Caserio-Alves F, Brito J, Araujo AE (2007). Liver hemangioma: common and uncommon findings and how to improve the differential diagnosis. Eur Radiol.

[6] Shepherd NA, Lee G (1983). Solitary necrotic nodules of the liver simulating hepatic metastases. J Clin Pathol.

[7] Berry CL (1985). Solitary 2 “necrotic nodule” of the liver: a probable pathogenesis. J Clin Pathol.

[8] Makhlouf HR, Ishak KG (2002). Sclerosed hemangioma and sclerosed hemangioma of the liver: a comparative clinicopathologic and immunohistochemical study with emphasis on the role of mast cells in their histogenesis. Liver.

[9] Miyamoto S, Oshita A, Daimaru Y, Sasaki M, Ohdan H, Nakamitsu A (2015). Hepatic Sclerosed Hemangioma: a case report and review of the literature. BMC Surg.

[10] Hida T, Nishie A, Tajima T, Taketomi A, Aishima S, Honda H (2010). Sclerosed hemangioma of the liver: possible diagnostic value of diffusion-weighted magnetic resonance imaging. Jpn J Radiol.

[11] Miyata T, Beppu T, Baba H (2018). Hepatic sclerosed hemangioma with special attention to diffusion-weighted magnetic resonance imaging. Surg Case Rep.

[12] Ho CL, Chen S, Yeung DW, Cheng TK (2007). Dual-tracer PET/CT imaging in evaluation of metastatic hepatocellular carcinoma. J Nucl Med.

[13] Yamashita Y, Shimada M, Taguchi K, Gion T, Hasegawa H, Utsunomiya T (2000). Hepatic sclerosing hemangioma mimicking a metastatic liver tumor: report of a case. Surg Today.

[14] Mathieu D, Rahmouni A, Vasile N (1994). Sclerosed liver hemangioma mimicking malignant tumor at MR imaging: pathologic correlation. J Magn Reson Imaging.

[15] Ryder SD (2003). Guidelines for the diagnosis and treatment of hepatocellular carcinoma in adults. Gut.

